# Theoretical insights into seawater metal ion binding on β-d-ribose: site selectivity, thermodynamics, and electronic properties

**DOI:** 10.1039/d6ra02408d

**Published:** 2026-07-03

**Authors:** Ayoub Kanaani, Mahmood Akbari, Alireza Vakili

**Affiliations:** a UNESCO-UNISA-iThemba LABS/NRF Africa Chair in Nanoscience and Nanotechnology, College of Graduate Studies, University of South Africa (UNISA) Muckleneuk Ridge, P.O. Box 392 Pretoria South Africa makbari@tlabs.ac.za; b School of Chemistry, Damghan University Damghan 36716-41167 Iran a.kanaani@yahoo.com; c Department of Geology, Faculty of Science, Ferdowsi University of Mashhad Mashhad Iran

## Abstract

Metal ion contamination of seawater poses growing environmental and public health challenges, driving demand for sustainable, selective adsorbents. Here, we employ density functional theory (DFT) at the (M06-2X/6-311++G(d,p) level with implicit aqueous solvation (PCM) to investigate the adsorption of seawater metal ions (Na^+^, K^+^, Ca^2+^, Mg^2+^, Fe^2+^, Zn^2+^) onto β-d-ribose. Among six evaluated coordination sites, Site 5 consistently exhibited the strongest binding for all cations, attributable to its highly nucleophilic oxygen atoms. The binding affinity follows the order Fe^2+^>> Zn^2+^ > Mg^2+^ >> Ca^2+^ > K^+^ ≥ Na^+^, consistent with the cation charge density and M–O bond lengths. NBO second-order perturbation energies *E*^(2)^ (kcal mol^−1^) further reflect this trend: Fe^2+^ (120.30) > Zn^2+^ (87.27) > Mg^2+^ (64.94) > Ca^2+^ (24.84) > Na^+^ (19.38) > K^+^ (5.39). Divalent cations, particularly Fe^2+^, exhibit significantly stronger interactions and greater charge-transfer stabilization compared to monovalent ions, as corroborated by AIM analysis showing the highest electron density at the bond critical point (*ρ*) for the Fe^2+^⋯Ri complex. Global reactivity descriptors further confirm the electronic stability and relative softness of the resulting complexes. Overall, β-d-ribose shows promise as a bio-based scaffold for selective ion capture with potential applications in desalination and environmental remediation.

## Abstract 2

β-d-ribose captures seawater metal ions through its most nucleophilic Site 5. Binding is strongest for divalent ions, following Fe^2+^ >> Zn^2+^ > Mg^2+^ >> Ca^2+^ > K^+^ ≥ Na^+^.

## Graphical abstract

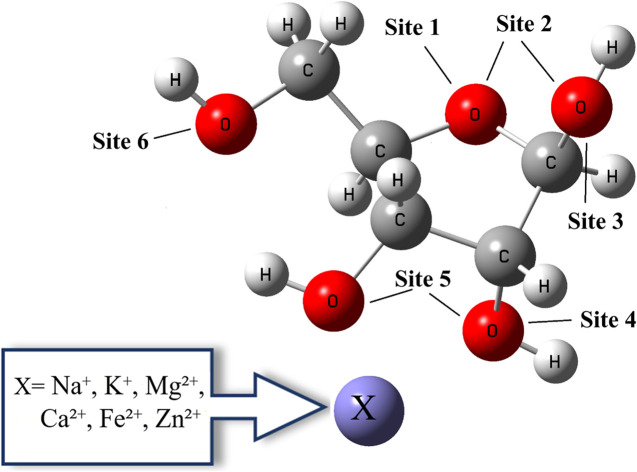

## Introduction

The contamination of seawater by metal ions, ranging from abundant alkali metals (Na^+^, K^+^) to alkaline earth metals (Ca^2+^, Mg^2+^) and transition metals (Fe^2+^, Zn^2+^), is a growing environmental concern with significant implications for drinking water quality, soil fertility, and aquatic ecosystems.^1,2^ Traditional remediation strategies, such as ion exchange, chemical precipitation, and membrane filtration, are often hindered by high operational costs, secondary waste generation, and limited selectivity.^3,4^ Consequently, there is an urgent need for sustainable, low-cost adsorbents capable of efficient and selective metal ion uptake.

Biopolymers derived from renewable resources have emerged as promising “green” adsorbents due to their biodegradability, tunable functionality, and natural abundance.^5,6^ In particular, polysaccharides and their monomeric units, such as glucose, glucosamine, and *N*-acetylglucosamine, have been extensively studied for metal-binding applications.^7,8^ Recent DFT investigations on d-glucose and related sugar monomers revealed that divalent cations bind significantly more strongly than monovalent ions, with oxygen-rich donor sites governing adsorption selectivity.^9^ More broadly, molecular-level insights into sugar–metal coordination are relevant to the rational development of polysaccharide hydrogels and polysaccharide-derived coordination frameworks for water treatment, catalysis, and biosensing applications.^10–13^

β-d-Ribose (Ri), the furanose monosaccharide backbone of RNA and numerous coenzymes, has not yet been explored as a model adsorbent for metal ions.^10^ Compared to the six-membered ring of glucose, the five-membered furanose ring of ribose presents a distinct spatial arrangement of hydroxyl groups and the ring oxygen, which may afford different coordination geometries and binding affinities toward metal cations.^11^ A systematic DFT study of ribose–metal interactions can therefore reveal how ring size and substituent orientation influence adsorption strength and site specificity, offering new molecular design principles for sugar-derived adsorbent materials.

In this work, we employ hybrid DFT methods to investigate the adsorption of Na^+^, K^+^, Ca^2+^, Mg^2+^, Fe^2+^, and Zn^2+^ onto β-d-ribose across six coordination sites, optimized using the Polarizable Continuum Model (PCM) to simulate the aqueous environment. Binding was characterized through M–O distances, interaction energies (Δ*E*), and Gibbs free energies (Δ*G*); donor–acceptor stabilization was probed *via* natural bond orbital (NBO) second-order perturbation energy (*E*^(2)^); charge redistribution was visualized using molecular electrostatic potential (MESP) maps; and complex stability and reactivity were assessed through HOMO–LUMO gaps and global reactivity descriptions, including hardness, softness, and electrophilicity indices.^12,13^ To our knowledge, this is the first comprehensive DFT investigation of β-d-ribose as a metal-binding scaffold. The results reveal how its unique furanose ring geometry modulates cation affinity relative to six-membered sugars and provide molecular-level insights to guide the design of ribose-based adsorbents, including polymeric networks and functional membranes, for selective ion capture in water treatment, resource recovery, and biomedical applications.

## Materials and methods

**Fig. 1:**
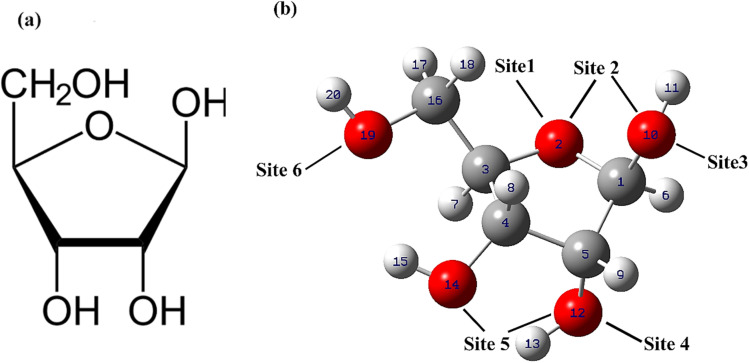
(a) Schematic structure of β-d-ribose and (b) optimized structure of six proposed metal-binding sites.

The adsorption of alkali and alkaline-earth cations (X^+^ = Na^+^, K^+^; X^2+^ = Ca^2+^, Mg^2+^, Fe^2+^, Zn^2+^) onto β-d-ribose (Ri) was investigated using density functional theory (DFT). Six distinct coordination sites on the Ri scaffold (Sites 1–6; Fig. 1a and b) were identified from preliminary conformational scans and used as starting points for all metal-substrate complexes.

All quantum-chemical calculations were performed using Gaussian 09 W.^14^ Geometry optimizations and harmonic frequency analyses of the bare Ri monomer and each X⋯Ri complex were carried out at the M06-2X/6-311++G(d,p) level of theory.^15^ The M06-2X functional was selected for its proven proficiency in treating non-covalent interactions, thermochemistry, and metal–ligand coordination, all of which are critical for the accurate calculation of adsorption energies.^7,15–17^ The 6-311++G(d,p) basis set was employed for all atoms.^18,19^ This triple-zeta split-valence basis set, augmented with diffuse and polarization functions on both non-hydrogen and hydrogen atoms, ensures sufficient orbital flexibility to describe charge-transfer pathways. The Polarizable Continuum Model (PCM) was applied throughout to account for the desolvation energy of the metal ions, providing a more realistic representation of the aqueous environment than vacuum-phase models and ensuring that the calculated trends reflect the thermodynamics of the adsorption process in solution.

Vibrational frequency calculations confirmed that all optimized structures are true minima on the potential energy surface, as evidenced by the absence of imaginary frequencies, and provided the thermal corrections necessary for enthalpy and free energy evaluations.

For the Fe^2+^⋯Ri complex, the most stable electronic configuration was determined to be the high-spin state with spin multiplicity 5 (*S* = 2; electron configuration: d_*z*2_^2^ d_*xy*_^1^ d_*yz*_^1^ d_*xz*_^1^ d_*x*^2^–*y*^2^_^1^). The spin multiplicities of 1 and 3 were found to lie 80.4 and 49.3 kcal mol^−1^ higher in energy, respectively, confirming the high-spin ground state.

To account for the seawater environment in which the target ions naturally occur, single-point energy calculations and frequency evaluations were repeated using the integral equation formalism variant of the PCM (IEF-PCM) water model^20^ at the same level of theory, enabling assessment of solvation effects on both structure and energetics.

Natural bond orbital (NBO) analysis was performed with NBO 3.1 as implemented in Gaussian 09 W, to quantify donor–acceptor stabilization energies, *E*^(2)^, arising from second-order perturbation interactions between lone-pair orbitals on Ri and vacant acceptor orbitals on the metal-center.^21^ Atoms-in-Molecules (AIM) analysis was carried out using AIM 2000 software^22^ to determine the electron density at the bond critical points of X⋯Ri interactions.

For each X⋯Ri complex, the interaction energy (Δ*E*), enthalpy (Δ*H*), and Gibbs free energy (Δ*G*) were computed according to the following thermochemical cycle:

1 Δ*Y*(Ri–X) = *Y*(Ri–X) − [*Y*(Ri) + *Y*(X)]

where *Y* represents the total electronic energy (*E*), enthalpy (*H*), or Gibbs free energy (*G*) evaluated at 298 K and 1 atm. Negative values of Δ*E*, Δ*H*, or Δ*G* indicate exothermic and thermodynamically spontaneous adsorption, respectively.

Frontier molecular orbital energies (*E*_HOMO_, *E*_LUMO_) were extracted from single-point calculations on the optimized geometries. The following DFT-based global reactivity descriptors were evaluated:^22,23^

2 Band gap energy: *E*_g_ = *E*_LUMO_ − *E*_HOMO_

3 Chemical hardness: *η* = 1/2(*E*_LUMO_ − *E*_HOMO_)

4 Chemical potential: *µ* = 1/2(*E*_LUMO_ + *E*_HOMO_)

5 Electrophilicity index: *ω* = *µ*^2^/2*η*

6 Global softness: *S* = 1/2*η*

7 Electronegativity: *χ* = −*µ*

These descriptors were calculated at the M06-2X/6-311++G(d,p) level to assess the relative stability and reactivity of the X⋯Ri complexes. Lower hardness (*η*) and higher softness (*S*) are associated with increased chemical reactivity, while higher electrophilicity index (*ω*) reflects a greater propensity to accept electron density from nucleophilic species.

Molecular Electrostatic Potentials (MESP) maps were generated for both the bare Ri molecule and each X⋯Ri complex in the aqueous phase. The MESP provides a visual representation of regions susceptible to nucleophilic attack (negative potential, red), electrophilic attack (positive potential, blue), or neither (neutral potential, green). All molecular visualization and surface renderings were prepared using GaussView 5.0.

## Results and discussion

### Adsorption site identification and stability

Geometry optimizations of β-d-ribose (Ri) and its cation complexes (X⋯Ri; X = Na^+^, K^+^, Mg^2+^, Ca^2+^, Fe^2+^, Zn^2+^) in the aqueous phase at the M06-2X/6-311++G(d,p) level revealed six distinct coordination sites on the ribose scaffold (Sites 1–6). These sites correspond to different oxygen-containing functional groups capable of binding the cations. The molecular structure of ribose with the proposed binding sites is shown in Fig. 1a and b, and the optimized X⋯Ri complex geometries at each site are provided in Fig. S1.

Among all sites, Site 5 emerged as the most stable adsorption site for every cation. In fact, Site 5 emerged as the global minimum configuration for every cation studied. The relative energies of the remaining sites, referenced to Site 5 (set to zero), are comparatively modest for the monovalent cations, only ∼2.1–3.5 kcal mol^−1^ higher for Na^+^ and K^+^, but increase substantially for the divalent cations: 5.8–8.0 kcal mol^−1^ for Mg^2+^, 9.3–12.2 kcal mol^−1^ for Ca^2+^, 12.6–31.2 kcal mol^−1^ for Fe^2+^, and 8.2–11.4 kcal mol^−1^ for Zn^2+^ (Fig. 1b). This pronounced energy gap underscores the exceptional thermodynamic stability of Site 5 complexes, particularly for divalent cations, and is consistent with the stronger electrostatic and charge-transfer interactions these ions engage in with the nucleophilic oxygen donors at this site. Nonetheless, all six sites remain viable binding sites, as even the highest-energy alternative site is within ∼31 kcal mol^−1^ of the minimum for Fe^2+^ (and much closer for the others), indicating multiple potential adsorption modes. These findings closely parallel those reported for d-glucose under analogous conditions,^7^ suggesting that the ribose molecule offers a cation binding environment broadly comparable to glucose in terms of site preference and relative affinity.

### X⋯O bond lengths

**Fig. 2:**
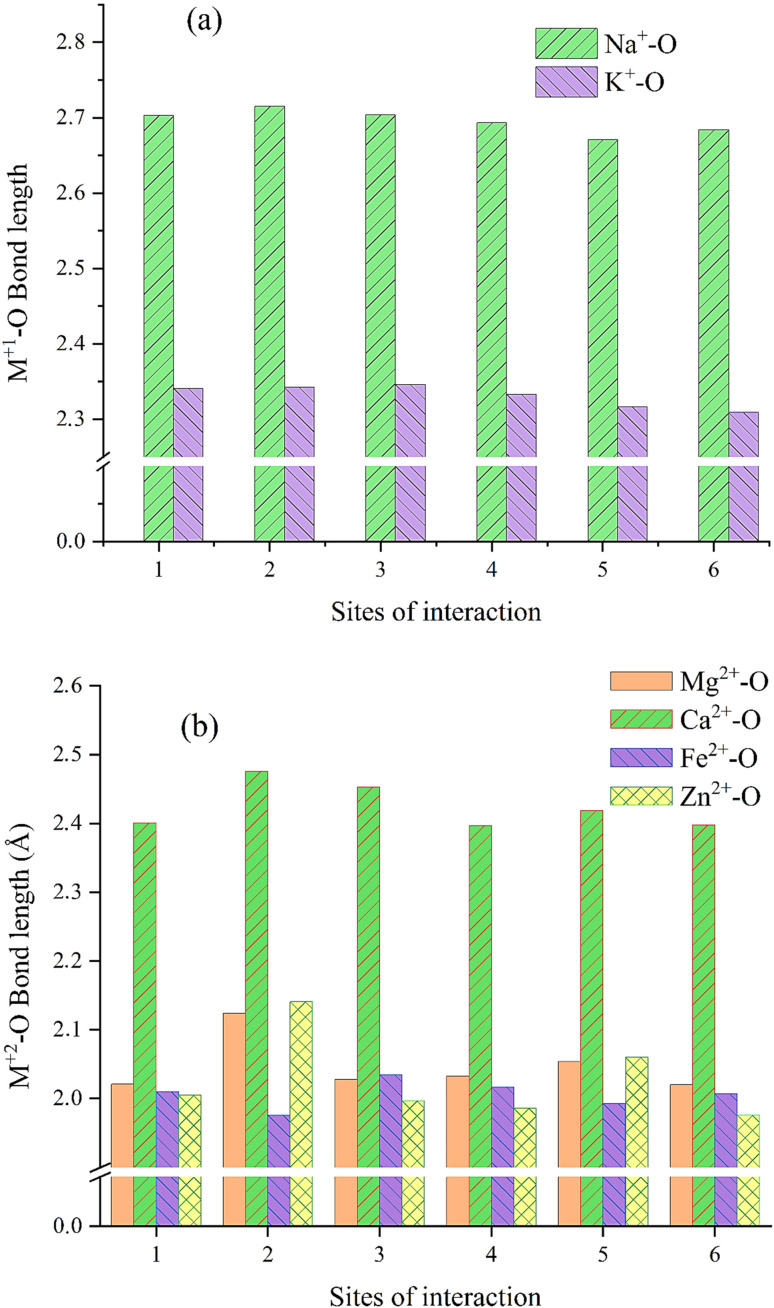
Average metal–oxygen bond lengths (Å) (a) for monovalent (b) and divalent cations at each ribose coordination site.

The strength and character of cation binding were further examined through analysis of the metal–oxygen bond lengths in each complex. The averaged X⋯O distances across the six coordination sites for all cations are depicted in Fig. 2a and b and listed in Table S1. As expected from electrostatic considerations, monovalent cations form relatively longer bonds with ribose oxygen donors, whereas divalent cations yield shorter bond lengths owing to their higher charge density. Specifically, Na^+^–O distances span 2.309–2.442 Å across the six sites, and K^+^–O distances fall in the range 2.674–2.715 Å. In contrast, the corresponding ranges for Ca^2+^, Mg^2+^, Fe^2+^, and Zn^2+^ are 2.397–2.476 Å, 2.020–2.124 Å, 1.976–2.035 Å, and 1.976–2.141 Å, respectively. These data demonstrate that Mg^2+^ and Fe^2+^ form by far the shortest X⋯O bonds among the cations studied, reflecting a combination of strong electrostatic attraction and partial covalent character in their interactions with the ribose oxygen donors. Although Ca^2+^ also binds more tightly than the monovalent ions, as evidenced by its shorter bond lengths, its interactions are weaker than those of Mg^2+^, consistent with its larger ionic radius and correspondingly lower charge density.

Notably, the average X⋯O bond lengths for the monovalent cations Na^+^ and K^+^ remain nearly constant across all six coordination sites, in contrast to the more pronounced site-dependent variation observed for the divalent cations. This site-independence indicates that Na^+^ and K^+^ exhibit little geometric discrimination between the available binding environments on the ribose scaffold, consistent with their more diffuse electrostatic interactions and lower sensitivity to the local oxygen donor arrangement.

All coordination bond lengths fall within the ranges expected for metal–oxygen interactions of this type, confirming that each of the six proposed sites can accommodate the respective cation without significant geometric strain. The overall bond length trends are fully consistent with the binding energy data discussed below, supporting the well-established correlation between shorter X⋯O distances and stronger adsorption. Furthermore, the absolute bond length values obtained here are in good agreement with those reported for the analogous cation complexes of d-glucose,^7^ underscoring that ribose and glucose, despite differing in ring size, exhibit broadly similar metal–oxygen coordination geometries when interacting with alkali and alkaline-earth metal ions.

### Interaction energies

All cation–ribose complexes have negative interaction energies (Δ*E*), confirming that adsorption is an exothermic and energetically favorable process at each of the six coordination sites. The binding (adsorption) energies of the Na^+^ and K^+^ complexes are less negative than those of the divalent cation complexes, as expected given the lower charge of monovalent ions. Within the monovalent series, K^+^ shows slightly more exothermic binding than Na^+^ across all sites, suggesting a marginally stronger interaction with the ribose oxygen donors. Among the divalent cations, Mg^2+^ binding is roughly twice as exothermic as that of Ca^2+^, and the most negative interaction energies are observed for Fe^2+^. The overall binding affinity follows the order Fe^2+^ >> Zn^2+^ > Mg^2+^ >> Ca^2+^ > K^+^ ≥ Na^+^, which is consistent with the relative charge densities of the cations and the M–O bond length trends discussed above.

The absolute adsorption energies on ribose are somewhat smaller in magnitude than those reported for the analogous glucose complexes, likely because the five-membered furanose ring of ribose offers a slightly less optimal coordination geometry and fewer simultaneous binding contacts than the six-membered pyranose ring of glucose.^7^ Nevertheless, the differences are modest, and the interaction energies on ribose are of the same order of magnitude as those on glucose,^7^ indicating that both monosaccharides possess comparable affinity for these metal cations.

**Fig. 3:**
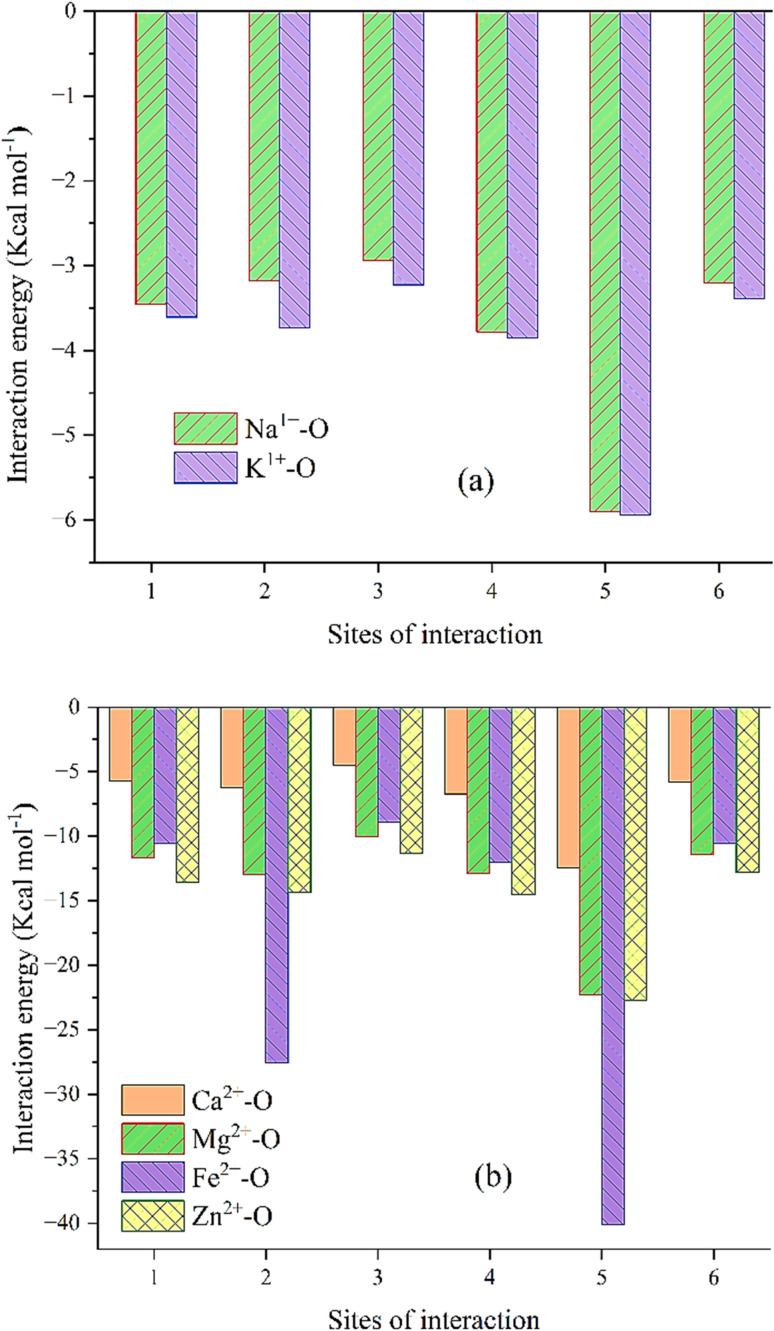
Interaction energies (Δ*E*, kcal mol^−1^) of (a) monovalent and (b) divalent cations adsorbed at six ribose sites.

Site 5 stands out as the most energetically favorable coordination site by a considerable margin for all six cations studied (Fig. 3). Quantitatively, the binding energy at Site 5 is approximately twice that at the next most favorable site, reflecting the uniquely favorable combination of nucleophilic oxygen donors and optimal coordination geometry available at this position. This pronounced site selectivity is observed consistently across both monovalent and divalent cations, identifying Site 5 as the dominant adsorption site on the β-d-ribose scaffold. The interaction energy trend at Site 5 is further corroborated by other binding descriptors: Site 5 complexes simultaneously exhibit the largest NBO donor–acceptor stabilization energies *E*^(2)^ and the most negative Gibbs free energies Δ*G* among all six sites (Tables 1 and 2). Notably, across all cations and sites, the complex with the highest *E*^(2)^ invariably corresponds to the most negative Δ*G*, and in every case, this is the Site 5 complex, demonstrating the internal consistency of the energetic, electronic, and thermodynamic descriptors employed. Taken together, the interaction energy analysis unambiguously identifies Site 5 as the predominant adsorption site on β-d-ribose for all cations examined, in full agreement with findings on the analogous glucose system.^7^

**Table 1:** The calculated *E*^(2)^ values (kcal mol^−1^) for LP(O) → LP*(X) at various sites of X⋯substrate complexes [X = Na^+^, K^+^, Mg^2+^, Ca^2+^, Fe^2+^, and Zn^2+^]

	Site 1	Site 2	Site 3	Site 4	Site 5	Site 6
Na^+^	4.57	17.26	3.88	3.97	19.38	3.30
K^+^	1.98	4.55	1.63	1.84	5.39	1.35
Mg^2+^	17.91	48.16	20.05	22.66	64.94	22.66
Ca^2+^	7.80	22.08	7.84	11.05	24.84	8.87
Fe^2+^	40.36	93.27	42.72	48.12	120.30	42.51
Zn^2+^	35.13	58.55	33.75	44.89	87.27	40.25

**Table 2:** Enthalpies (Δ*H*) and Gibbs free energies (Δ*G*) changes (kcal mol^−1^) at various sites of X⋯substrate complexes [X = Na^+^, K^+^, Mg^2+^, Ca^2+^, Fe^2+^, and Zn^2+^]

	Site 1		Site 2		Site 3		Site 4		Site 5		Site 6	
	Δ*H*	Δ*G*	Δ*H*	Δ*G*	Δ*H*	Δ*G*	Δ*H*	Δ*G*	Δ*H*	Δ*G*	Δ*H*	Δ*G*
Na^+^	−2.61	−29.57	−2.14	−27.24	−2.03	−28.82	−2.84	−28.73	−4.69	−29.62	−2.12	−28.30
K^+^	−2.69	−28.88	−2.87	−29.37	−2.32	−29.08	−4.14	−27.95	−4.89	−30.14	−2.32	−28.44
Mg^2+^	−9.87	−33.94	−11.00	−34.35	−9.30	−31.53	−10.92	−34.96	−19.90	−43.15	−8.96	−32.96
Ca^2+^	−4.39	−29.73	−4.78	−28.71	−3.08	−28.04	−5.34	−30.72	−10.80	−35.12	−4.68	−30.96
Fe^2+^	−11.51	−34.24	−25.38	−47.49	−7.07	−30.72	−10.49	−34.83	−37.96	−60.91	−9.13	−32.76
Zn^2+^	−11.97	−36.08	−11.66	−33.31	−9.87	−34.01	−12.92	−36.83	−21.00	−44.48	−11.10	−35.39

### NBO second-order perturbation (*E*^(2)^) and atoms-in-molecules (AIM) analysis

NBO analysis is widely employed to examine bond interactions and intermolecular charge transfer (ICT) between orbitals.^24,25^ To elucidate the electronic factors governing cation binding, a natural bond orbital (NBO) second-order perturbation analysis was performed. This approach quantifies donor–acceptor interactions between filled orbitals on the ribose, principally the oxygen lone pairs, and the vacant acceptor orbitals of the metal cation, yielding the stabilization energy *E*^(2)^ associated with each donation event. The sum of these second-order stabilization energies provides an estimate of the total charge-transfer contribution to bonding in the complex. A higher *E*^(2)^ value reflects a stronger donor–acceptor interaction, arising from greater orbital overlap between the donor and acceptor, a larger interaction matrix element between the oxygen lone pair and the metal's empty orbitals, and more significant delocalization of electron density from the ribose oxygen into the metal centre, effectively contributing to the formation of a coordinate covalent bond.

Consistent with the interaction energy results, Site 5 complexes exhibit the largest *E*^(2)^ values among the six sites for every cation (Table 1), confirming that electron donation from ribose to the metal is most effective at this site. This is reflected in Site 5's combination of short X⋯O bonds, high binding energies, and strong donor–acceptor stabilization. Across the cation series, the magnitude of *E*^(2)^ follows the order Fe^2+^ > Zn^2+^ > Mg^2+^ > Ca^2+^ > Na^+^ > K^+^, indicating that Fe^2+^⋯ribose complexes show significantly greater charge-transfer stabilization than the corresponding complexes of any other cation studied. Divalent cations induce considerably larger *E*^(2)^ values than monovalent cations, owing to their higher ionic charge and smaller ionic radius, both of which intensify electrostatic attraction to the negatively polarized oxygen atoms of β-d-ribose and enhance polarization of the ribose electron cloud, thereby promoting greater charge transfer and more pronounced coordination interactions.

This behavior is physically expected: a divalent cation, by virtue of its higher positive charge and accessible low-lying empty orbitals, can accept electron density more readily and engage in stronger NBO delocalization with the substrate lone pairs than a monovalent ion of comparable size. As an illustrative example, the total second-order donation energy for Mg^2+^ at a given site is approximately an order of magnitude greater than that for Na^+^ and K^+^ at the same site, reflecting the markedly stronger Lewis acid character of the divalent ion. Collectively, the NBO results confirm that divalent cations form more robust, charge-transfer-enhanced bonds with ribose, whereas monovalent cations rely predominantly on weaker, diffuse electrostatic interactions. The NBO analysis thus provides a quantitative electronic basis for the observed binding energy trends and rationalizes the outstanding donor–acceptor stabilization at Site 5.

**Fig. 4:**
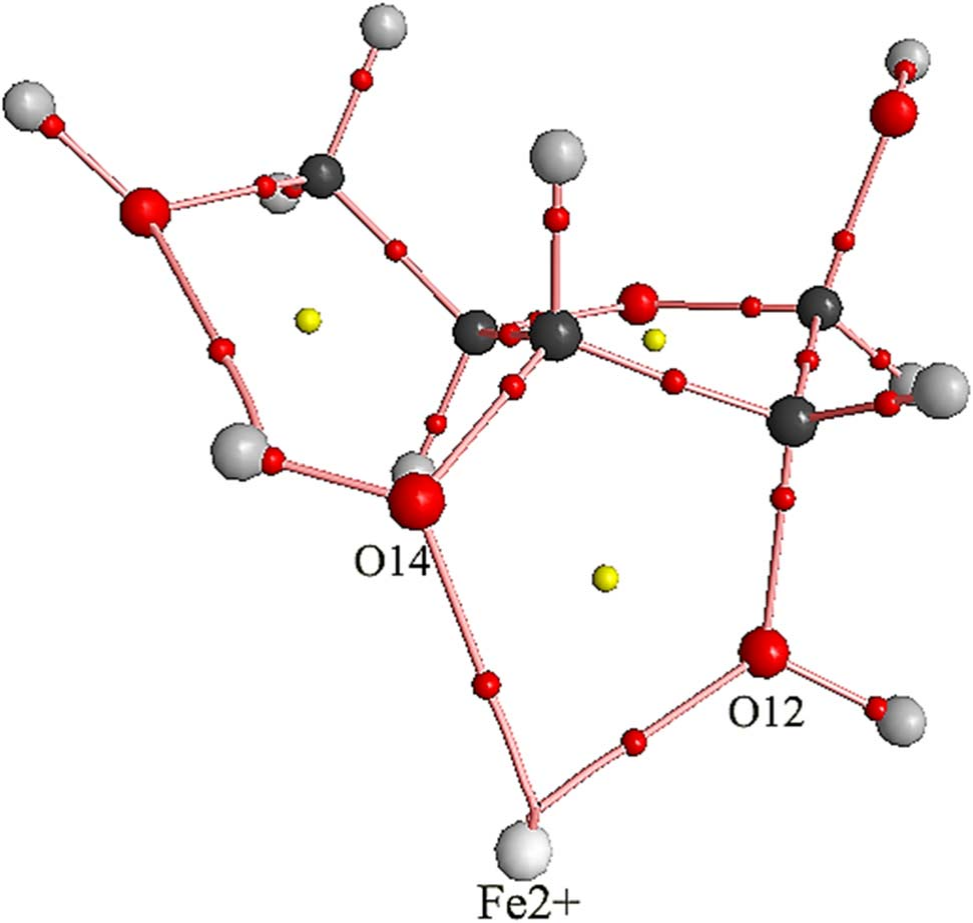
Molecular graphs of Fe^2+^⋯Ri, red points indicate the bond critical points (BCPs) between bonded atoms (color figure online).

Bader's atoms-in-molecules (AIM) analysis was performed to further characterize the nature of the X⋯Ri interactions at a topological level.^26,27^ A representative molecular graph of the X⋯Ri complex is shown in Fig. 4.

The AIM analysis yields topological parameters at the bond critical points (BCPs), the (3, −1) saddle points in the electron density, including the electron density *ρ*(*r*), the Laplacian of electron density ∇^2^*ρ*, the kinetic energy density *G*, the potential energy density *V*, and the total electronic energy density *H*; these are compiled in Table 3. The magnitudes of *ρ* and the ∇^2^*ρ* at the BCPs provide direct information on the nature and strength of each metal-ribose interaction. Positive values of the ratio −*G*/*V* exceeding unity [−*G*/*V* > 1] are diagnostic of closed-shell, non-covalent interactions between the X^*n*+^ ions and the heteroatom donors of the ribose scaffold. The *ρ* values compiled in Table 3 are appreciable across all complexes, indicating significant metal–ribose interaction in each case; notably, the Fe^2+^⋯Ri and Zn^2+^⋯Ri complexes exhibit the highest *ρ* values, consistent with their superior binding affinities established above.

**Table 3:** Electron density (*ρ*), Laplacian of the electron density (∇^2^*ρ*), kinetic energy density (*G*), potential energy density (*V*), and total electron energy density (*H*) in a.u. at BCPs of the most stable (Site 5) in M^*n*+^-substrate complexes by AIM analysis

Complex	BCP	*ρ*	∇^2^*ρ*	*G*	*V*	*H*	−*G*/*V*
Na^+^⋯Ri	Na⋯O14	0.046	0.092	0.079	−0.066	−0.013	1.195
	K⋯O12	0.041	0.079	0.067	−0.055	−0.012	1.213
K^+^⋯Ri	K⋯O14	0.019	0.022	0.019	−0.016	−0.003	1.195
	Mg⋯O12	0.018	0.021	0.018	−0.015	−0.003	1.204
Mg^2+^⋯Ri	Mg⋯O14	0.042	0.074	0.065	−0.055	−0.010	1.173
	Ca⋯O12	0.039	0.067	0.059	−0.050	−0.009	1.178
Ca^2+^⋯Ri	Ca⋯O14	0.034	0.042	0.037	−0.033	−0.004	1.125
	Fe⋯O12	0.032	0.039	0.035	−0.030	−0.004	1.135
Fe^2+^⋯Ri	Fe⋯O14	0.075	0.116	0.119	−0.121	0.002	0.980
	Zn⋯O12	0.072	0.114	0.116	−0.117	0.001	0.989
Zn^2+^⋯Ri	Zn⋯O14	0.051	0.113	0.096	−0.079	−0.017	1.218
	Zn⋯O12	0.043	0.076	0.066	−0.058	−0.011	1.138

To further validate the thermodynamic stability trends, the correlation between interaction energy (Δ*E*) and electron density (*ρ*) at the metal–oxygen BCPs was examined. A strong linear relationship was observed, wherein the higher binding affinities of the divalent cations Fe^2+^ and Zn^2+^ relative to the monovalent cations Na^+^ and K^+^ are mirrored by significantly higher *ρ* values at the corresponding BCPs. The uniformly positive Laplacian (∇^2^*ρ* > 0) at all BCPs confirms the closed-shell, predominantly electrostatic nature of the metal–ribose interactions across all coordination sites, consistent with the hard-soft acid-base (HSAB) framework applicable to these species. Where discrepancies arise between the magnitude of thermodynamic stability and the topology of individual bond critical points, these are attributable to the chelate effect: the cooperative engagement of multiple oxygen donor sites provides additional stabilization that is not fully reflected in any single BCP parameter considered in isolation.

### Thermodynamic spontaneity of adsorption (Δ*G*)

In addition to interaction energies, the thermodynamics of cation adsorption were assessed through computation of the Gibbs free energy change (Δ*G*) for each complex (Table 2). All calculated Δ*G* values are negative, confirming that the adsorption of Na^+^, K^+^, Ca^2+^, Mg^2+^, Fe^2+^, and Zn^2+^ onto β-d-ribose is thermodynamically spontaneous under aqueous-phase conditions. Even at the least favorable coordination site, the free energy of binding remains exergonic, while at Site 5, the Δ*G* is substantially more negative, reflecting a strong thermodynamic driving force for complexation. Consistent with the trends established above, Site 5 yields the most negative Δ*G* for every cation, further reinforcing its identity as the thermodynamically preferred coordination site on the ribose scaffold.

A pronounced contrast exists between the thermodynamic behavior of monovalent and divalent cations: the Δ*G* magnitudes for the divalent complexes are approximately four times greater than those for the Na^+^ and K^+^ complexes, highlighting the considerably stronger affinity of β-d-ribose for doubly charged cations. The trend in Gibbs free energies closely mirrors that of the interaction energies (Δ*E*), with the binding spontaneity decreasing in the order Fe^2+^ > Zn^2+^ > Mg^2+^ > Ca^2+^ > K^+^ > Na^+^, consistent with the charge density ranking established from the bond length and NBO analyses. The uniformly negative Δ*G* values across all cations and sites confirm that even the weakest complexation event, Na^+^ binding at the least favorable site, remains thermodynamically accessible, while the strongest, Fe^2+^ binding at Site 5, is associated with a substantial thermodynamic driving force. Collectively, these results demonstrate that β-d-ribose can spontaneously coordinate metal cations across a range of charge densities under aqueous conditions, with a marked thermodynamic preference for divalent ions, particularly Fe^2+^, highlighting its potential as a bio-based scaffold for selective metal ion capture.

### Molecular electrostatic potential (MESP) analysis

To examine how charge redistributes upon cation binding, molecular electrostatic potential (MESP) surface maps were generated for the isolated β-d-ribose molecule and each X⋯Ri complex; these are provided in Fig. S2 and S3 (SI). The MESP analysis also provides insight into the origin of Site 5's exceptional binding preference. Among all oxygen donor sites on ribose, the oxygen atoms constituting Site 5 carry the most negative partial charges in the free molecule, as evidenced by the deep red intensity at this site in the MESP map and corroborated by NBO atomic charge calculations (Fig. S2). This identifies Site 5 as inherently the most nucleophilic region of the ribose scaffold, and therefore the site most predisposed to attracting and coordinating positively charged metal ions. Upon cation binding at Site 5, the localized negative charge is substantially compensated, as reflected in the shift of the MESP coloration from deep red to blue, a visual signature of strong electrostatic interaction.

Fig. S3 presents the MESP maps of the most stable representative X⋯Ri complexes alongside that of the unbound ribose (Fig. S2) for comparison. In the free ribose, a strongly negative electrostatic potential (red regions) is concentrated around the hydroxyl oxygen atoms and the ring oxygen, consistent with their high electron density and intrinsic nucleophilicity. These electronegative regions correspond directly to the proposed cation binding sites. Following cation adsorption, the MESP undergoes a pronounced redistribution: the previously electron-rich oxygen sites become markedly less negative or acquire a slight positive character (green to blue coloration), particularly at the coordinated site. Across all six coordination sites of ribose, the intense red regions are substantially diminished upon complex formation, with the degree of color shift reflecting the extent of electrostatic interaction with the bound cation.

The MESP maps are thus consistent with significant charge redistribution upon complexation, suggesting that electron density is shifted from the oxygen lone pairs toward the metal center; this interpretation is further supported and quantified by the NBO donor–acceptor analysis presented above. The pronounced polarization exerted by the metal cation on the electron cloud of the ribose scaffold is clearly reflected in the electrostatic potential surfaces.

The superior binding affinity at Site 5 arises from a synergistic combination of structural and electronic factors. Structurally, Site 5 presents a bidentate coordination geometry involving two adjacent oxygen donors, the C4 hydroxyl oxygen and the ring oxygen, which together form a stable five-membered chelate ring with the metal ion. This geometry minimizes steric strain while maximizing orbital overlap between the donor lone pairs and the metal acceptor orbitals, providing an optimal coordination environment that is not replicated at any other site on the ribose scaffold. Electronically, the MESP analysis identifies Site 5 as the region of highest negative electrostatic potential on the free ribose, creating a preferential electrostatic hotspot for the approaching cation. Importantly, NBO second-order perturbation analysis confirms that the binding at Site 5 is not purely electrostatic in character: significant charge-transfer stabilization, quantified by the *E*^(2)^ energies associated with donor–acceptor interactions between the hydroxyl oxygen lone pairs [LP(O)] and the vacant metal acceptor orbitals [LP*(M)], is most pronounced at this site relative to all others. The magnitude of this covalent contribution to bonding is therefore the primary electronic factor that differentiates Site 5 from the remaining coordination sites on the ribose scaffold and accounts for its dominant role in metal ion capture.

### Global reactivity indices and electronic descriptors

The global reactivity descriptors of the X⋯Ri complexes, namely the HOMO–LUMO gap and the derived indices hardness (*η*), softness (*S*), chemical potential (*µ*), electronegativity (*χ*), and electrophilicity (*ω*), were evaluated to assess the electronic stability and chemical reactivity of each complex. The values of these descriptors, calculated from the frontier orbital energies of each optimized complex, are presented in Table S2. All six classes of complex exhibit a large HOMO–LUMO energy gap (*E*_g_ in the range 7.6–9.5 eV), corresponding to high global hardness and low global softness. A gap of this magnitude implies that the X⋯Ri complexes are electronically stable and relatively inert toward excitation and charge transfer, owing to the substantial energetic separation between the occupied and virtual molecular orbitals. Consequently, the complexes are resistant to polarization and redox processes, consistent with the general robustness expected of metal–sugar coordination complexes. The near-uniformity of the gap values across the Na^+^, K^+^, Ca^2+^, Mg^2+^, Fe^2+^, and Zn^2+^ complexes indicates that all possess comparable intrinsic electronic stability, confirming that coordination of either a monovalent or divalent cation does not significantly perturb the frontier orbital structure of the ribose scaffold.

Closer inspection of Table S2 reveals subtle but chemically meaningful differences in reactivity indices between the monovalent and divalent cation complexes. The Fe^2+^···Ri and Zn^2+^···Ri complexes exhibit slightly larger HOMO–LUMO gaps and correspondingly lower global softness than the remaining complexes, indicating that Fe^2+^ and Zn^2+^ binding render the system marginally harder, that is, less chemically reactive and less polarizable, relative to complexes formed with Mg^2+^, Ca^2+^, or the alkali metal cations. This trend can be rationalized in terms of the stronger polarizing power of Fe^2+^ and Zn^2+^: their greater ability to withdraw electron density from the ribose narrows the effective HOMO–LUMO gap relative to the less polarizing monovalent cations. Conversely, Na^+^ and K^+^ complexes exhibit the highest softness values in the series, consistent with the more diffuse, less directional electrostatic interactions these ions form with the ribose, which leave the frontier orbital energies of the scaffold comparatively unperturbed.

The electrophilicity index (*ω*), which quantifies the propensity of a species to accept electron density,^28^ varies appreciably across the cation series. Fe^2+^···ribose complexes exhibit the highest *ω* values (4.39–5.02 eV), Ca^2+^ and Mg^2+^ complexes display intermediate values (approximately 2.9–3.6 eV), and the Na^+^ and K^+^ complexes have the lowest *ω* (approximately 2.7 eV) (Table S2). The elevated electrophilicity of divalent–cation complexes reflects their stronger electron-accepting character: Fe^2+^, Zn^2+^, Mg^2+^, and Ca^2+^ complexes have a greater capacity to withdraw electron density from nucleophilic donors than their monovalent analogues. The electrophilicity index has been established in the literature as a useful descriptor of chemical and biological reactivity,^7,23^ with higher *ω* values correlating with greater potential reactivity toward biological nucleophiles and, in some cases, with increased biological activity or cytotoxicity.^7^ In this context, the Fe^2+^···ribose complex, which exhibits the highest *ω* in the series, possesses the greatest propensity for electrophilic interaction with biological nucleophiles, whereas the Na^+^ and K^+^ complexes, with the lowest *ω* values, are the least electrophilic and consequently the least reactive in a biochemical context. This pattern is consistent with that reported for cation adsorption on d-glucose,^7^ where divalent cation complexes similarly exhibited higher electrophilicity than their monovalent counterparts.

Taken together, the global reactivity descriptors indicate that the metal ion's hardness and softness relative to the adsorbent's donor sites are key determinants of binding strength and selectivity. Divalent transition metal ions such as Fe^2+^ and Zn^2+^, which behave as relatively soft Lewis acids, engage in greater covalent interaction and charge transfer with the oxygen donors of β-d-ribose, leading to stronger adsorption and enhanced selectivity relative to the harder alkali metal ions Na^+^ and K^+^. For the rational design of selective adsorbents, these findings underscore the importance of matching the hardness and softness of the adsorbent's functional groups to those of the target metal ions to maximize both binding affinity and specificity.

The global reactivity analysis collectively reinforces that all X⋯Ri complexes are electronically robust, characterized by large HOMO–LUMO gaps and high hardness, while divalent cation binding additionally imparts a moderately more reactive character, reflected in higher softness and electrophilicity, relative to monovalent cation binding. These electronic nuances may be consequential in applications where the secondary reactivity of the complex is relevant, such as in biological environments or in the design of stimuli-responsive adsorbent materials. In summary, the DFT results present a coherent and internally consistent picture: β-d-ribose coordinates metal cations robustly across multiple sites, with Site 5 as the dominant binding locus; binding strength and electrophilic character are markedly enhanced for divalent cations, in full agreement with prior findings on analogous glucose systems^7^ and with the predictions of hard-soft acid-base theory.

### Proposed modifications to enhance selectivity and efficiency

Based on the molecular-level insights gained from this study, the following structural modifications of β-d-ribose are proposed as promising directions for enhancing its ion capture selectivity and adsorption efficiency and are recommended for future experimental and computational investigation.

Substitution of hydroxyl groups with stronger donor functionalities represents the most direct route to enhanced metal binding. Amination, replacement of hydroxyl protons with amine groups (–NH_2_), introduces nitrogen as an additional Lewis base donor capable of participating in metal coordination. Thiolation, introduction of thiol groups (–SH), replaces the hard oxygen donors with the softer sulfur atom, thereby shifting selectivity toward softer metal ions such as Hg^2+^, Pb^2+^, and Cd^2+^ in accordance with HSAB principles. Conversion of hydroxyl groups to carboxylate functionalities (–COOH or –COO^−^) introduces more polarisable oxygen donors and generates negatively charged coordination sites upon deprotonation, enhancing electrostatic attraction toward cations across a range of charge densities.

Polymerization and scaffold engineering offer a complementary strategy for amplifying adsorption capacity. Grafting functionalized ribose units onto robust polymer backbones, such as chitosan, cellulose, or synthetic polymers, creates high-density arrays of binding sites in which cooperative, synergistic interactions between adjacent donor groups may enhance both capacity and selectivity. Alternatively, the construction of ribose-based dendrimers or hyperbranched structures would concentrate multiple binding sites within a confined spatial domain, promoting chelate-type binding and enabling steric discrimination between ions of differing sizes.

Steric and geometric tuning through position-specific functionalization provides a means of engineering binding pocket geometry. Introducing sterically demanding substituents at selected hydroxyl positions can create size-selective binding cavities that favor smaller ions while impeding access by larger ones. Incorporation of ligand groups with defined coordination preferences, for example, those favoring tetrahedral geometry for Zn^2+^ or octahedral geometry for Fe^2+^, may further enhance ion-specific selectivity.

Finally, the incorporation of ionizable functional groups such as carboxylic acids (–COOH) or amines (–NH_2_) would confer pH-responsive adsorption behavior. The protonation state of these groups, and hence their metal-binding affinity, can be modulated by solution pH, enabling selective loading of target ions under acidic conditions and their controlled release under basic conditions, a feature of considerable practical value in regenerable adsorbent systems.

## Conclusion

This study presents a comprehensive density functional theory investigation of the adsorption of monovalent (Na^+^, K^+^) and divalent (Ca^2+^, Mg^2+^, Fe^2+^, Zn^2+^) metal ions onto β-d-ribose, providing the first systematic molecular-level characterization of this biologically and environmentally relevant monosaccharide as a metal-binding scaffold. Geometry optimizations at the M06-2X/6-311++G(d,p) level with PCM aqueous solvation identified six distinct coordination sites on the ribose scaffold, of which Site 5, characterized by the highest local nucleophilicity and an optimal bidentate coordination geometry, emerged as the thermodynamically preferred binding locus for all six cations examined.

Divalent cations consistently exhibit substantially stronger adsorption than monovalent ions across all metrics employed, including interaction energies (Δ*E*), Gibbs free energies (Δ*G*), M–O bond lengths, and NBO second-order perturbation energies *E*^(2)^. The overall binding affinity follows the order Fe^2+^ >> Zn^2+^ > Mg^2+^ >> Ca^2+^ > K^+^ ≥ Na^+^, directly reflecting the charge density of the cations. The uniformly negative Δ*G* values confirm that adsorption is thermodynamically spontaneous for all cation–ribose combinations under aqueous conditions, with divalent cation complexes exhibiting free energies of binding approximately four times more favorable than those of the monovalent analogues. AIM topological analysis corroborates these trends using electron density values at the metal–oxygen bond critical points, with Fe^2+^···Ri and Zn^2+^···Ri complexes exhibiting the highest *ρ* values in the series.

MESP analysis reveals pronounced charge redistribution upon complexation, consistent with the shift of electron density from the ribose oxygen lone pairs toward the metal center and rationalizes the exceptional preference for Site 5 due to its uniquely negative electrostatic potential. Global reactivity descriptors reveal that all X⋯Ri complexes are electronically robust, characterized by large HOMO–LUMO gaps (7.6–9.5 eV) and high global hardness, while the electrophilicity index (*ω*) is most pronounced for the divalent cation complexes, particularly Fe^2+^, consistent with their stronger Lewis acid character and greater charge-transfer capacity.

Collectively, these results establish β-d-ribose as a promising bio-based scaffold for selective metal ion capture, with inherent thermodynamic and electronic selectivity for divalent over monovalent cations. The molecular-level insights provided here, regarding the role of site nucleophilicity, coordination geometry, charge density, and donor–acceptor orbital interactions in governing adsorption selectivity, offer a rational foundation for the design of ribose-derived adsorbent materials, including functionalized polymers, hydrogels, and membrane systems, for applications in seawater desalination, heavy metal remediation, and resource recovery.

## Conflicts of interest

The authors declare that they have no known competing financial interests or personal relationships that could have appeared to influence the work reported in this paper.

## Supplementary Material

RA-016-D6RA02408D-s001

## Data Availability

All data generated or analysed during this study are included in this published article (and its supplementary information (SI) files). Supplementary information is available. See DOI: https://doi.org/10.1039/d6ra02408d.
