# Supplementary material for: Theoretical insights into seawater metal ion binding on β-d-ribose: site selectivity, thermodynamics, and electronic properties
Source: RSC Adv. 2026 Jul 3;16(35):35200–9. doi: 10.1039/d6ra02408d (PMC13329669; doi:10.1039/d6ra02408d)
Supplement: RA-016-D6RA02408D-s001 [file RA-016-D6RA02408D-s001.pdf]

Supplementary Information (SI) for RSC Advances.
This journal is © The Royal Society of Chemistry 2026

Supplementary Information

Theoretical Insights into Metal Ion Binding on *β*-D-Ribose: Site Selectivity, Thermodynamics, and Electronic Properties

Figure S1: Optimized structures of β-*D*-ribose with adsorbed Na^+ (a), K^+ (b), Mg^{2+} (c), Ca^{2+} (d), Fe^{2+} (e), Zn^{2+} (f), and at six different coordination sites, along with their relative energies (in kcal/mol) calculated at the M06-2X/6-311++G(d,p) level in aqueous phase.

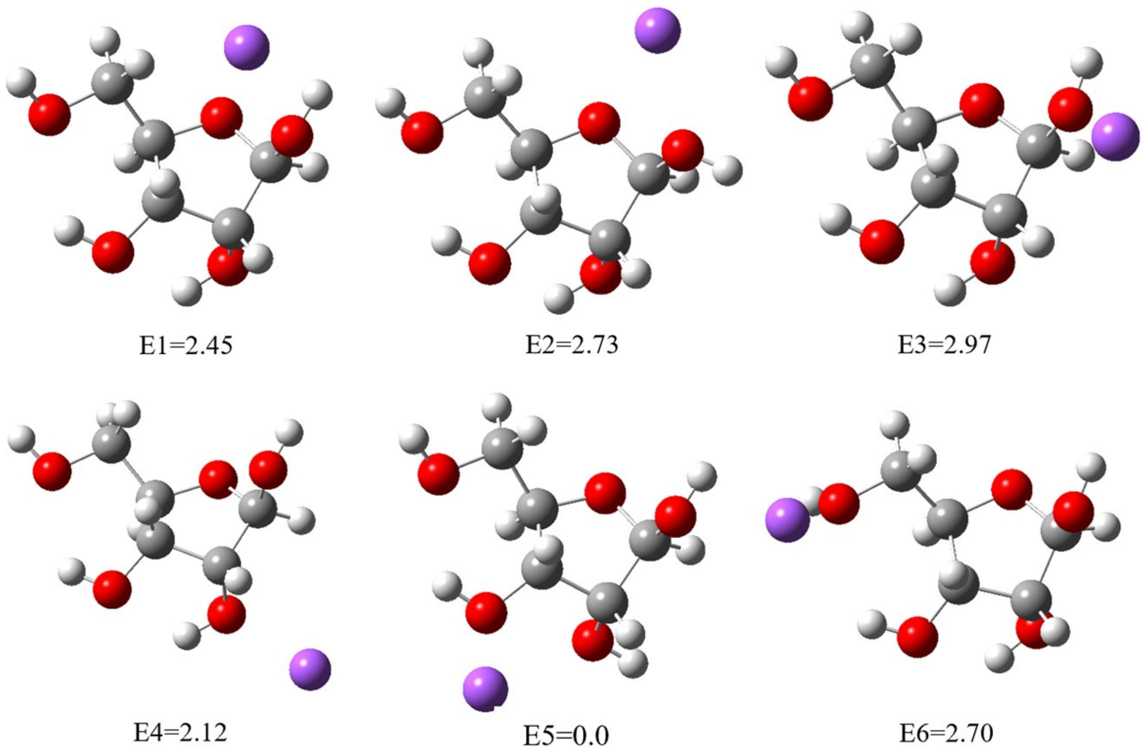

a) Optimized structures of the adsorption of sodium ion (Na^+) on ribose.

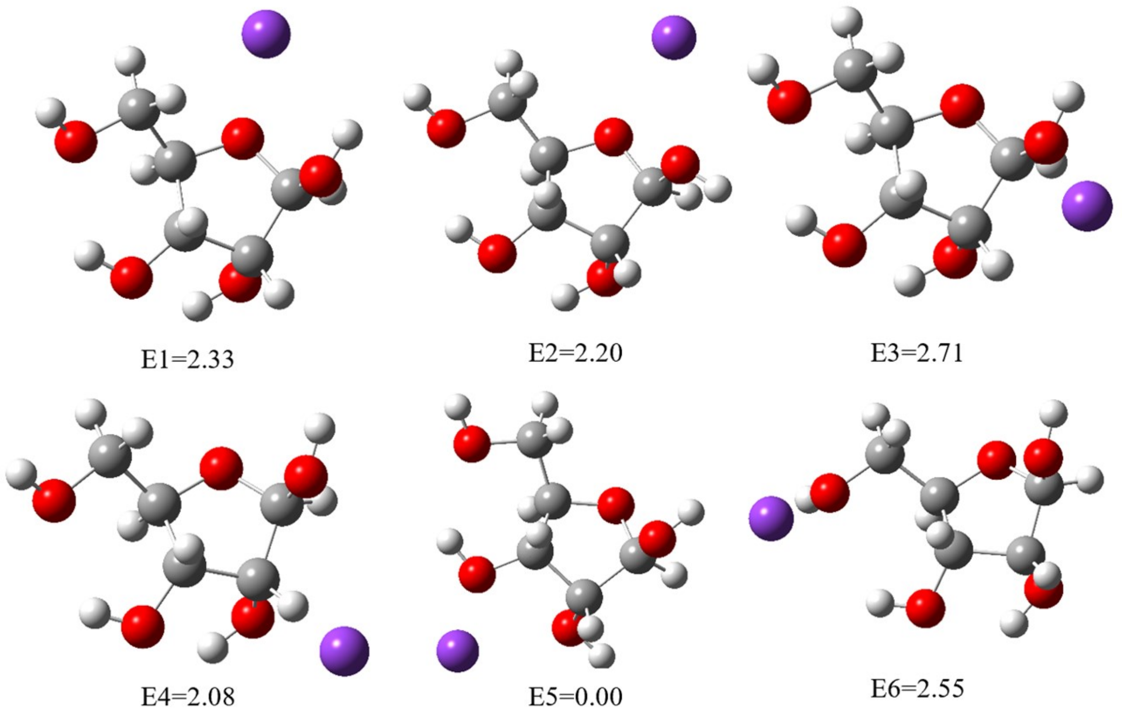

b) Optimized structures of adsorption of potassium ion (K^+) on ribose.

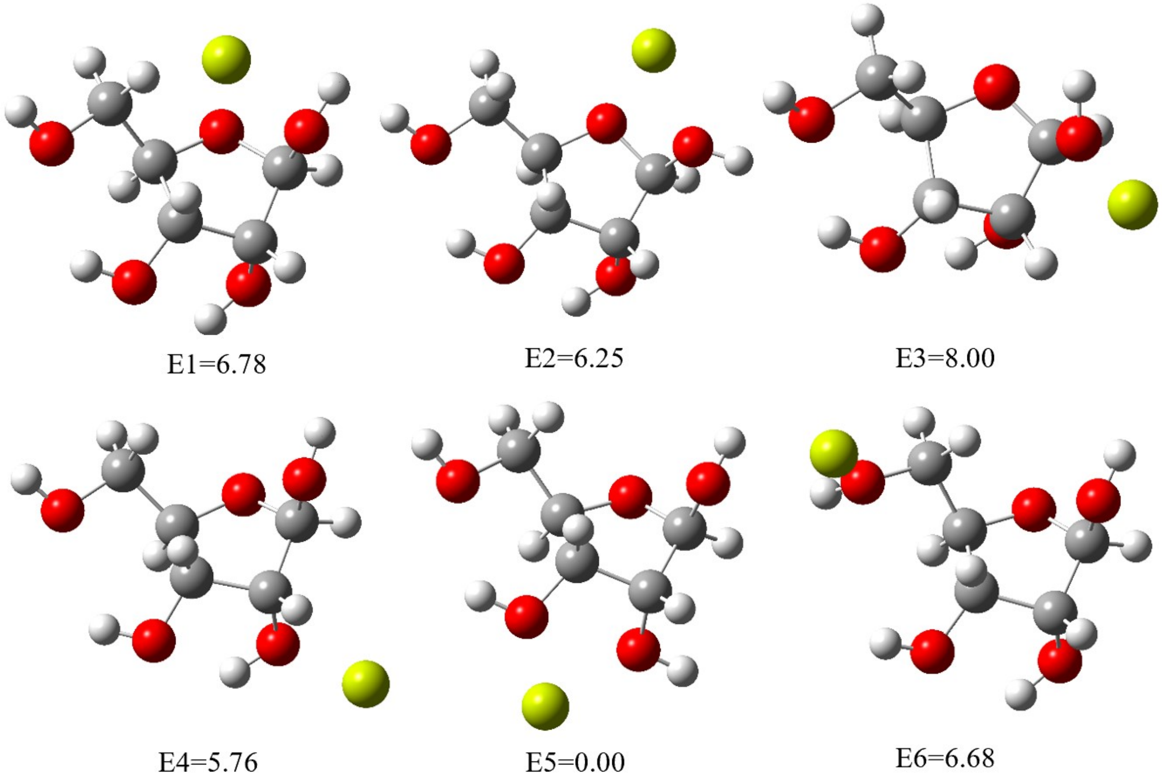

c) Optimized structures of adsorption of magnesium ion (Mg^{2+}) on ribose.

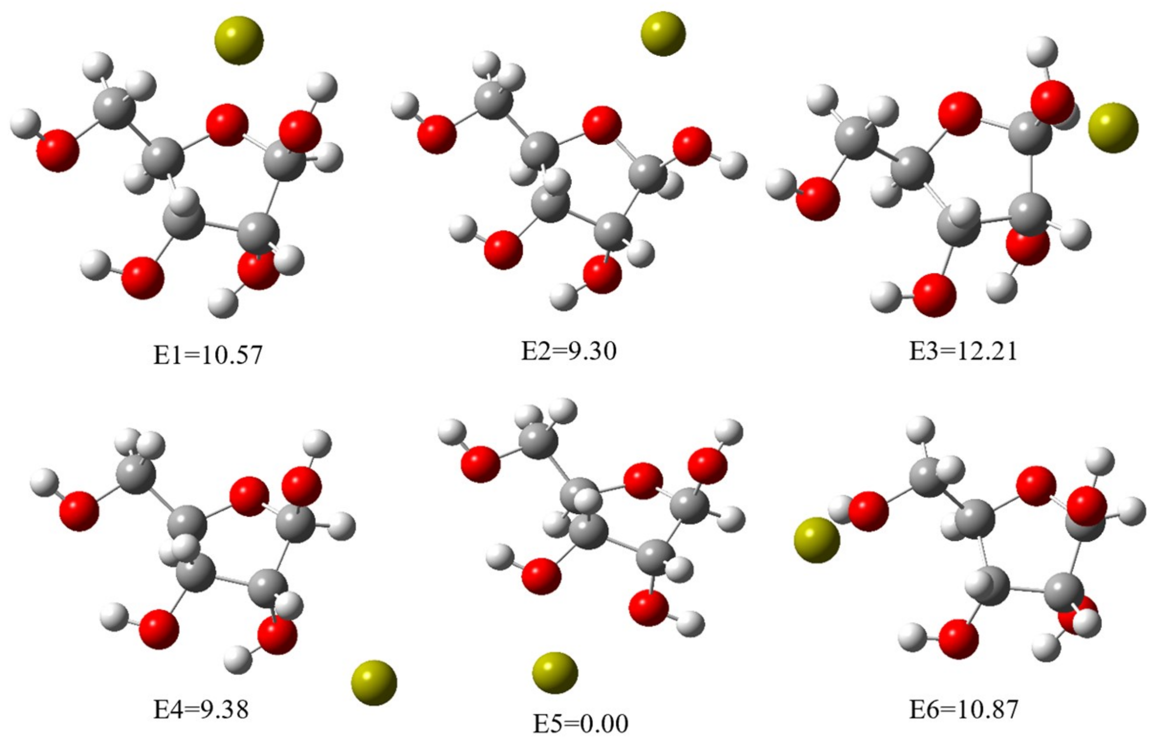

d) Optimized structures of the adsorption of calcium ion (Ca^{2+}) on ribose.

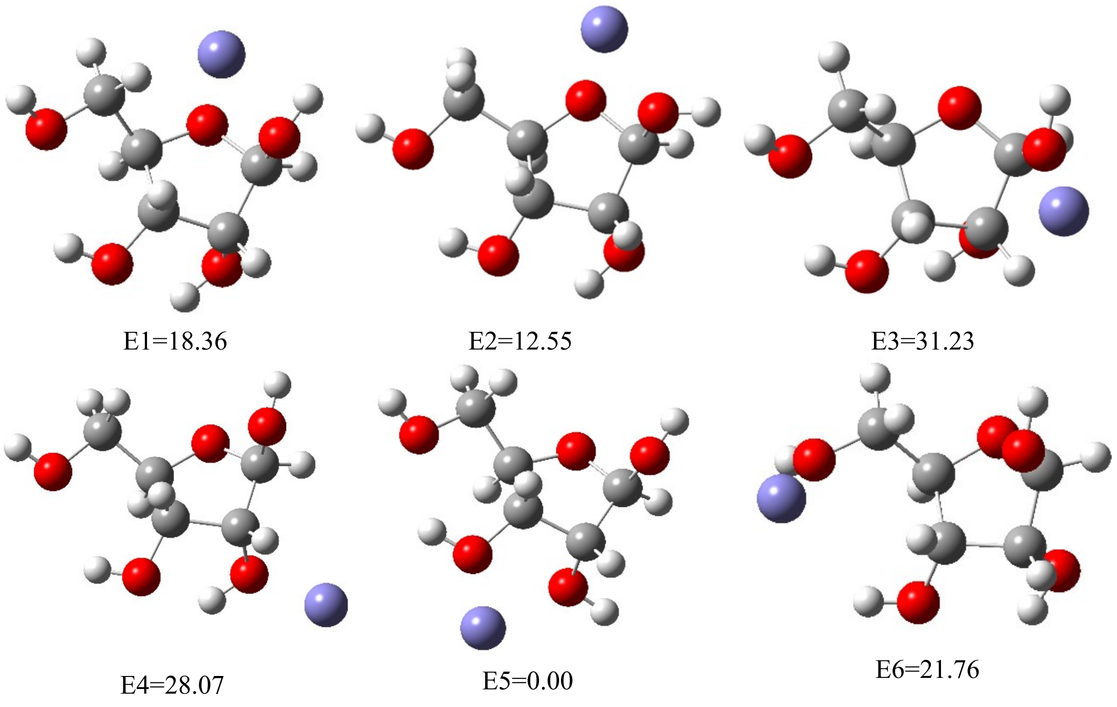

e) Optimized structures of adsorption of iron ion (Fe^{2+}) on ribose.

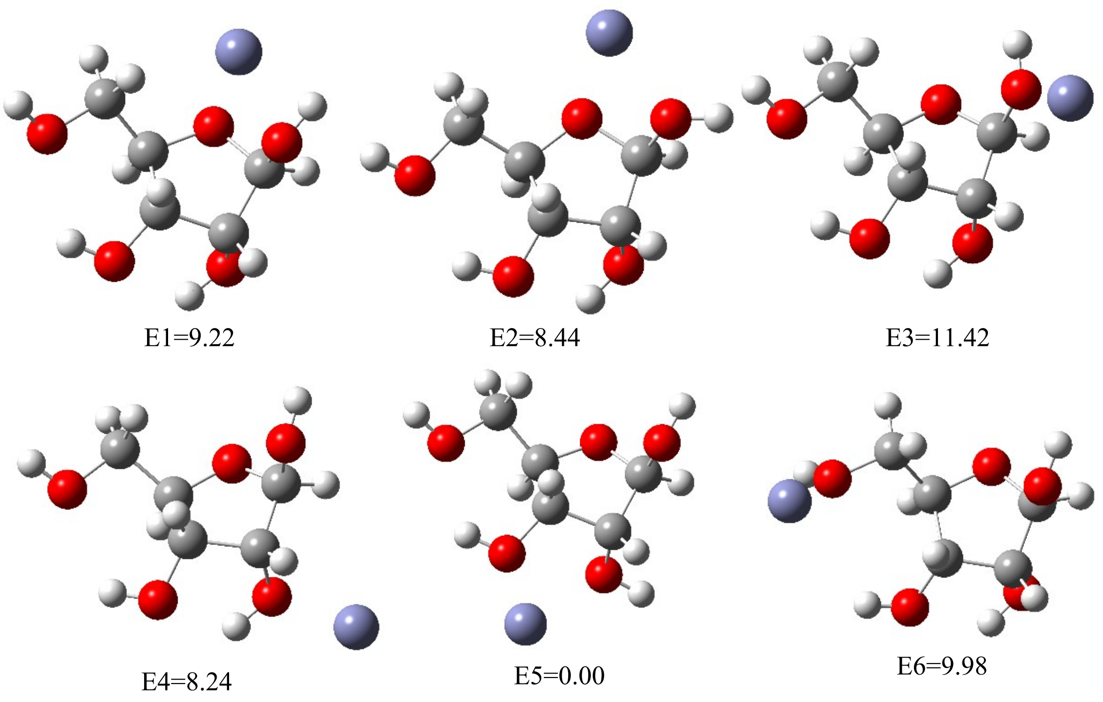

f) Optimized structures of adsorption of zinc ion (Zn^{2+}) on ribose.

Figure S2. MESP of substrates before adsorption of cation and NBO atomic charges on oxygen atoms (atom numbering can be found in Fig. 1) at different ribose sites, highlighting Site 5 as the most negatively charged and nucleophilic region.

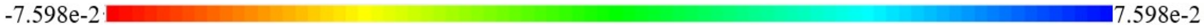

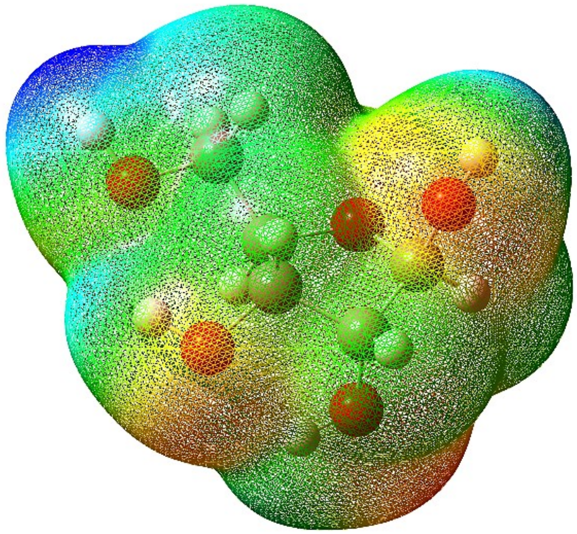

Atom number	Charge	Electric Potential
O2	-0.208	-22.327
O10	-0.268	-22.321
O12	-0.240	-22.338
O14	-0.309	-22.325
O19	-0.350	-22.308

Figure S3. Molecular electrostatic potential (MESP) surfaces of the representative most stable Ri...X complexes.

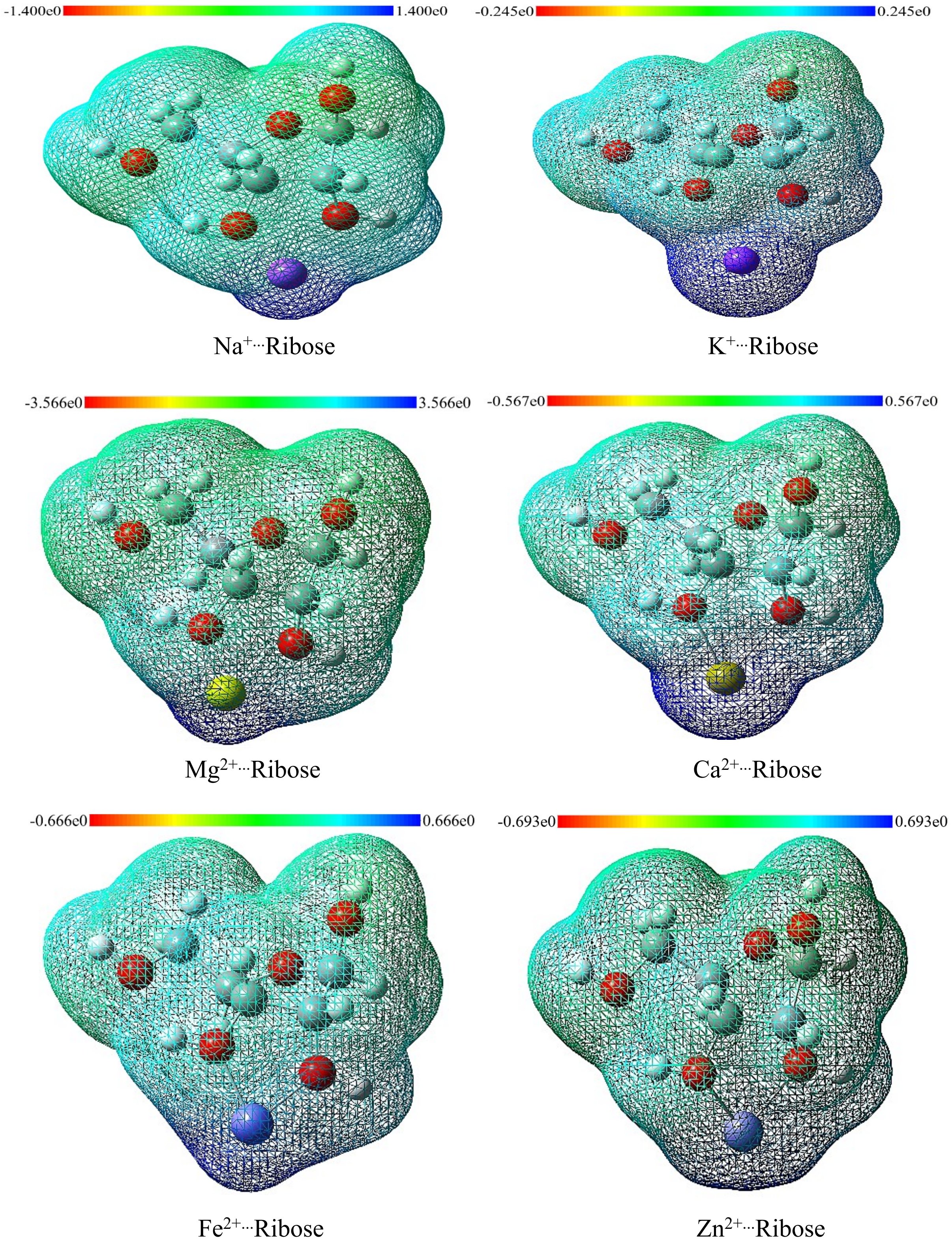

Table S1. Average metal···oxygen bond lengths (in Å) for Na^{+}, K^{+}, Ca^{2+}, Mg^{2+}, Fe^{2+}, and Zn^{2+} at each of the six ribose coordination sites.

	Na^{+}	K^{+}	Ca^{2+}	Mg^{2+}	Fe^{2+}	Zn^{2+}
1	2.341	2.703	2.401	2.021	2.010	2.005
2	2.442	2.715	2.476	2.124	1.976	2.141
3	2.346	2.704	2.453	2.028	2.035	1.997
4	2.313	2.683	2.397	2.033	2.017	1.986
5	2.346	2.691	2.419	2.054	1.993	2.060
6	2.309	2.674	2.398	2.020	2.009	1.976

Table S2. Calculated energy gap and global reactivity parameters (in eV) of the X-substrate complexes in different sites by at M06-2X.6-311++G (d,p) basis set [X= Na^+, K^+, Ca^{2+}, Mg^{2+}, Fe^{2+}, and Zn^{2+}].

Na^+	HOMO	LUMO	E_g	μ	η	S	χ	ω
1	-9.734	-0.351	9.383	-5.043	4.691	0.107	5.043	2.710
2	-9.792	-0.339	9.453	-5.065	4.727	0.106	5.065	2.714
3	-9.712	-0.364	9.348	-5.038	4.674	0.107	5.038	2.715
4	-9.702	-0.341	9.361	-5.021	4.680	0.107	5.021	2.694
5	-9.748	-0.352	9.396	-5.050	4.698	0.106	5.050	2.714
6	-9.633	-0.400	9.233	-5.017	4.617	0.108	5.017	2.726
K^+	HOMO	LUMO	E_g	μ	η	S	χ	ω
1	-9.710	-0.379	9.331	-5.044	4.665	0.107	5.044	2.727
2	-9.768	-0.358	9.410	-5.063	4.705	0.106	5.063	2.724
3	-9.691	-0.388	9.303	-5.04	4.651	0.107	5.04	2.730
4	-9.688	-0.370	9.318	-5.029	4.659	0.107	5.029	2.714
5	-9.711	-0.370	9.341	-5.04	4.670	0.107	5.04	2.720
6	-9.591	-0.416	9.175	-5.004	4.588	0.109	5.004	2.728
Mg^{2+}	HOMO	LUMO	E_g	μ	η	S	χ	ω
1	-10.000	-1.199	8.801	-5.599	4.401	0.114	5.599	3.562
2	-10.105	-1.026	9.079	-5.565	4.540	0.110	5.565	3.411
3	-9.953	-1.209	8.744	-5.581	4.372	0.114	5.581	3.562
4	-9.951	-1.184	8.767	-5.568	4.383	0.114	5.568	3.536
5	-10.117	-0.986	9.131	-5.551	4.566	0.110	5.551	3.375
6	-9.720	-1.247	8.473	-5.484	4.237	0.118	5.484	3.549
Ca^{2+}	HOMO	LUMO	E_g	μ	η	S	χ	ω
1	-9.885	-0.543	9.342	-5.214	4.671	0.107	5.214	2.91
2	-9.956	-0.500	9.456	-5.228	4.728	0.106	5.228	2.89
3	-9.853	-0.568	9.285	-5.211	4.643	0.108	5.211	2.924
4	-9.856	-0.532	9.324	-5.194	4.662	0.107	5.194	2.893
5	-9.972	-0.518	9.454	-5.245	4.727	0.106	5.245	2.910
6	-9.710	-0.601	9.109	-5.156	4.555	0.110	5.156	2.918
Fe^{2+}	HOMO	LUMO	E_g	μ	η	S	χ	ω
1	-10.028	-2.316	7.712	-6.172	3.856	0.130	6.172	4.939
2	-10.185	-2.072	8.113	-6.129	4.057	0.123	6.129	4.630
3	-9.987	-2.375	7.612	-6.181	3.806	0.131	6.181	5.019
4	-9.995	-2.299	7.696	-6.147	3.848	0.130	6.147	4.909
5	-10.205	-1.882	8.323	-6.043	4.162	0.120	6.043	4.388
6	-9.813	-2.316	7.497	-6.064	3.749	0.133	6.064	4.905

Zn^{2+}	HOMO	LUMO	E_g	μ	η	S	χ	ω
1	-10.027	-2.170	7.857	-6.099	3.928	0.127	6.099	4.734
2	-10.130	-1.971	8.159	-6.051	4.080	0.123	6.051	4.487
3	-9.974	-2.176	7.798	-6.075	3.899	0.128	6.075	4.732
4	-9.985	-2.130	7.856	-6.057	3.928	0.127	6.057	4.671
5	-10.159	-1.820	8.339	-5.989	4.170	0.120	5.989	4.301
6	-9.805	-2.187	7.618	-5.996	3.809	0.131	5.996	4.720
